# The Field of Science

**Published:** 1893-12-16

**Authors:** 


### THE FIELD OF SCIENCE.

From St. Louis we hear that a new system of ambulances has been applied to the electric railways which run through the town. The springs of these new auxiliaries are so constructed that no unnecessary jar or movement is felt by the invalid during the transit. Three large hospitals and the principal dispensary of the town are situated on the route through which the cars pass, and this fresh supplement to the already existing ambulance provision of the city will certainly prove a valuable one.

The Worthing sewerage question, which for some weeks past has proved itself of so particularly involved a nature, has at length been solved. The scheme of work for this overhauling is to cost the inhabitants about £36,000, but it cannot efficiently be carried out at a lower figure. The engineer proposes a new system of drainage in the southern portion of the town, from west to east, which will be ventilated by shafts and planned to discharge its contents during the four hours eastward flow of the tide in each twelve hours.

Faraday's investigation on the effect of the electric current on a solution of chloride of magnesium is the basis of an experiment by M. Hermite, which it is expected will prove of great practical value. His system is based upon the electrolysis of sea-water, and the electric current decomposes the chloride of magnesium whilst the chloride of sodium serves as a conductor. The result is a liquid disinfectant of great power, almost odourless, and leaving no residuum when used for the purpose of flushing. The solid sewage is instantaneously consumed, as well as all other organic matter. It destroys with equal certainty anaërobic and aërobic microbes. To the former class oxygen is the primal requirement, and as this chloride deprives them of that they become instantly extinct. The aërobic microbes are destroyed with equal rapidity. The corrosive action of the gas consumes them. Sea-water is the cheapest method for forming this disinfectant, but chloride of magnesium can be substituted. At Havre the Municipality, alarmed by the cholera advance, have furthered M. Hermite's experiments to the utmost, and the necessary tanks for making large quantities of the disinfectant have been constructed at a central depot, where also the necessary electrical machinery has been installed. The disinfectant is distributed throughout the town by pipes similar to the ordinary water system, and can be laid on to houses to be used for flushing all drains and waste water pipes. Where it is used there can be no escape of sewer gas, and all microbes are destroyed.

And there are numerous striking indications that not only in France, but in foreign countries, the authorities are becoming alive to the importance of M. Hermite's system of electrical sanitation. We learn this on the authority of one who actually was an eye-witness to the almost miraculous transformation, exhibited at Havre, of sewage into the inoffensive, odourless, and transparent yellow-tinted liquid which it becomes under this process. In France, the municipality of Brest is at the present moment preparing a practical trial of this purifying process, and before the end of December the English colony at Nice will have an opportunity of witnessing practical experiments of the Hermite system to be made at Brest at the cost of the municipality. We understand that the majority of Paris Councillors are in favour of a probing trial in the same direction. French bacteriologists have tested the sewage, which has been submitted to them after its electrical treatment, but no trace of any of the microbes so fatal to the human race could be discovered in it. The treatment of sewage by electricity was tried some time ago at Salford as an experiment, and at present it is being treated experimentally by the London County Council at Crossness. At seaports, as at Rouen and Havre, it seems to be regarded as entirely successful when applied to sewage in places where sea-water is readily attainable. No work appears to have been published on the subject at present, but we hope before long to be in a position to give our readers an exhaustive account of this system, which seems to be full of promise.

